# Cryoneurolysis as a Novel Treatment for Spasticity, Associated Pain and Presumed Contracture

**DOI:** 10.1177/27536351241285198

**Published:** 2024-09-30

**Authors:** Paul Winston, Daniel Vincent

**Affiliations:** 1 Division of Physical Medicine and Rehabilitation, University of British Columbia, Victoria, BC, Canada; 2 Canadian Advances in Neuro-Orthopedics for Spasticity Consortium, Kingston, ON, Canada; 3 Division of Anesthesia, University of British Columbia, Victoria, BC, Canada

Cryoneurolysis for spasticity and associated pain is an innovation of a decades-old pain technique. Initially described in 1961, liquid nitrogen was used at temperatures of −190°C. There has been a global resurgence of using cryogens at temperatures below −100°C, which allows for reversible neurolysis. At these temperatures, Wallerian degeneration or secondary axonotmesis occurs, which provides for the endoneurium, perineurium and epineurium to remain intact, permitting nerve regeneration at a rate of 1 to 2 mm per day.^1^ In 2018, we introduced mini-invasive percutaneous ultrasound (US) guided cryoneurolysis as a longer-lasting treatment option for spasticity, adapting the sensory nerve-targeted procedure to motor and sensorimotor nerves.

This pathway began in 2017 based on French and Belgian physicians who used landmark-guided anesthetic diagnostic motor nerve blocks (DNB) with lidocaine to identify what muscles contribute to spasticity and differentiate them from true contracture. The term *reducible deformity* is given to a muscle group that appears shortened or rigid and lengthens after a DNB. We created a curriculum of US-guided DNB to make them fast and efficient with e-stimulation. The addition of US meant that all nerves became targets, the sensorimotor trunk, motor branches to individual muscles or intramuscular nerve targets. We theorized that cryoneurolysis could be adapted to motor nerves using the machine created by Lloyd at Oxford at temperatures of −60°C using CO2 as the driving gas.

One 1998 case report on cryoneurolysis of an obturator nerve branch existed.^2^ In 2018, we performed our first motor nerve targets, the musculocutaneous nerve branches to brachialis and biceps, the radial nerve to the brachioradialis and the tibial nerve trunk and branches to the gastrocnemius and soleus. We have since published numerous guides to US targeting, from the shoulder to the foot, with multiple case reports and case series with figures and videos that show both the technique and case outcomes.^3^ By incorporating a demonstration of the challenging spastic anatomy and localization required, the goal is to create a global teaching syllabus while comprehensive studies are completed. Nerve-targeted procedures aim not just to reduce tone but to increase range of motion and improve power.^4^ The prescreening of patients with multiple DNB also represents a paradigm shift. The patient is shown what the outcome will likely look like before making an informed decision.

Advantages of cryoneurolysis include the absence of a maximal dose, which allows for far more muscles to be treated, including all muscles from a nerve trunk. Many muscles treated with botulinum toxin are off-label, depending on the country of practice. Neither phenol nor alcohol have on-label indications for spasticity and lack studies and are typically avoided for larger sensorimotor trunks. Chemical neurolysis leads to tissue destruction and vessel thrombosis. Cryoneurolysis at temperatures of −88°C used in spasticity, only destroys the neuron and spares the surrounding tissue. The heat sink of blood vessels protects their integrity as cryoneurolysis is often performed with the probe adjacent to blood vessels. Spasticity is frequently accompanied with pain, either at the tight resting position or end range, and it may lead to tissue breakdown.^1^ Cryoneurolysis is an established pain technique; adding sensory and mixed sensorimotor targets, such as the suprascapular or superficial fibular nerve or genicular nerves can offer multiple therapeutic benefits. Targeting the ubiquitous spastic claw hand contracture is possible via the sensorimotor ulnar and median nerve trunks to target contracture and pain.

We published on the first 113 patients recruited for 3 studies and 277 treated nerves.^5^ This was the first-ever paper dedicated to the side effects of cryoneurolysis and the first to address motor and mixed nerves. About 96.75% of nerve treatments had no pain or dysesthesias beyond treatment. Nine treatments had dysesthesia, 7 of which were in mixed sensorimotor nerves. Only 5 required any treatment of oral medication or corticosteroid injection. Few had pain or numbness beyond 3 months. Since publication, the DNB has been used to ensure that patients have no sensory change, and patients who report immediate nerve pain may undergo an additional cycle of treatment to remove any residual pain-generating sensory axons. Only motor targets will be chosen if a disturbance is present and not a sensorimotor trunk.

The published case literature and published abstracts from ongoing studies have shown that sustained benefits from a single treatment are maintained at the 1-year follow-up and further prolonged in case reports.^4^ Cryoneurolysis for spasticity has been performed on children from age 3 years and nonagenarians. The treatment can be painful with larger needles and catheter placement and the cramping burning sensation as nerves are destroyed, causing immediate muscle fasciculations. Pediatric sedation clinics are now available in our Canadian centre and in Atlanta, USA, with further USA centres to begin shortly. Other analgesic treatments include nitrous oxide gas, methoxyflurane (Penthrox), oral medications and sedatives and topical preparations can be offered to patients.

Since our 2018 introduction, cryoneurolysis for spasticity has been adopted in multiple centres in Canada, USA, Luxembourg, UK, France, Denmark and Spain. A training education platform of publications, international presentations and hands-on workshops has been the cornerstone of knowledge translation. The studies for cryoneurolysis generally have longer-term follow-ups than other spasticity treatments, which are 1 year.^5^ Thus, as the data is being collected, hands-on demonstration shows the immediate effect of cryoneurolysis with demonstrative, easily seen outcomes. Our centre currently has 6 studies on the upper and lower extremities. Additionally, studies in France comparing neurectomy versus cryoneurolysis and botulinum toxin versus cryoneurolysis in the UK and Luxembourg and a large multi-centre sham-controlled study in the USA are all entering the treatment phase. The initial financial analysis to be published will outline the cost savings of this technique that will allow cryoneurolysis to be adapted globally at a large cost savings from current dominant techniques. Our current equipment (iovera, Pacira Bioscinces, NJ, USA) costs approximately $500 USD for a single treatment, with most patients averaging less than 1 treatment per year. Globally, patients with spasticity have a severe lack of access to care. An affordable, cheaper, lasting treatment is needed for global implementation and study.
